# Transmitter Diversity Gain Technique Aided Irregular Channel Coding for Mobile Video Transmission

**DOI:** 10.3390/e23020235

**Published:** 2021-02-18

**Authors:** Nasru Minallah, Khadem Ullah, Jaroslav Frnda, Korhan Cengiz, Muhammad Awais Javed

**Affiliations:** 1 Department of Computer Systems Engineering, University of Engineering and Technology Peshawar, Peshawar 25000, Pakistan; n.minallah@uetpeshawar.edu.pk (N.M.); 14pwcse1224@uetpeshawar.edu.pk (K.U.); 2 Department of Quantitative Methods and Economic Informatics, Faculty of Operation and Economics of Transport and Communications, University of Zilina, 010 26 Zilina, Slovakia; 3 Department of Electrical-Electronics Engineering, Trakya University, 22030 Edirne, Turkey; korhancengiz@trakya.edu.tr; 4 Department of Electrical and Computer Engineering, COMSATS University Islamabad (CUI), Islamabad 45550, Pakistan; awais.javed@comsats.edu.pk

**Keywords:** IrRegular Convolutional Codes (IRCCs), IRCC-SP-DSTS

## Abstract

The reliable transmission of multimedia information that is coded through highly compression efficient encoders is a challenging task. This article presents the iterative convergence performance of IrRegular Convolutional Codes (IRCCs) with the aid of the multidimensional Sphere Packing (SP) modulation assisted Differential Space Time Spreading Codes (IRCC-SP-DSTS) scheme for the transmission of H.264/Advanced Video Coding (AVC) compressed video coded stream. In this article, three different regular and irregular error protection schemes are presented. In the presented Regular Error Protection (REP) scheme, all of the partitions of the video sequence are regular error protected with a rate of 3/4 IRCC. In Irregular Error Protection scheme-1 (IREP-1) the H.264/AVC partitions are prioritized as A, B & C, respectively. Whereas, in Irregular Error Protection scheme-2 (IREP-2), the H.264/AVC partitions are prioritized as B, A, and C, respectively. The performance of the iterative paradigm of an inner IRCC and outer Rate-1 Precoder is analyzed by the EXtrinsic Information Transfer (EXIT) Chart and the Quality of Experience (QoE) performance of the proposed mechanism is evaluated using the Bit Error Rate (BER) metric and Peak Signal to Noise Ratio (PSNR)-based objective quality metric. More specifically, it is concluded that the proposed IREP-2 scheme exhibits a gain of 1 dB $E_{b}/N_{0}$ with reference to the IREP-1 and $E_{b}/N_{0}$ gain of 0.6 dB with reference to the REP scheme over the PSNR degradation of 1 dB.

## 1. Introduction

The advent of diverse video applications and services increases the need for a highly efficient network, providing massive connectivity, reliable, and low latency communication, along with higher channel capacity. The future network has to be a combination of different technologies and intend to be efficiently managed, optimized, and integrated for diverse applications and networking scenarios, extending the battery lifetime of a low powered devices as well as providing a very fast and high data rate connectivity to the user with higher mobility [1]. Challenges still exist in providing higher data rates, portability, mobility, quality of service, security to end-user, and higher connectivity in wireless communication [2]. The limitations on the data rate are due to the limited available bandwidth, level of the signal, and the quality of the channel [3].

Furthermore, the end-user needs to transmit high definition videos on the correlated Rayleigh fading channel. Reducing the error rate on such a type of channel is an extremely a challenging task when compared to the wired communication channels, e.g., coaxial cable, fiber, microwave, etc. In the wired communication channel, reducing the error rate from $10^{-2}$ to $10^{-3}$ may be obtained by increasing 1 or 2 dB SNR. On the contrary, the same cannot be achieved in the next generation by increasing the transmission power or bandwidth, as it is contrary to the requirements of the next generation systems. Therefore, it is very important to mitigate the error rate without any further increase in the power or sacrifice in bandwidth [4]. In the Rayleigh fading channel, due to the destructive addition of multi-path propagation signals, the Signal to Noise Ratio (SNR) of the transmitted signal sometimes occurred under deep fade for which the receiver cannot reliably decode and estimate the exact transmitted signal. The diversity technique is used, in which multiple replicas of the same signal are transmitted from multiple transmitting antennas while assuming that one of the replicas has to be a sufficient SNR for decoding. An issue arises as to transmitting multiple copies with minimum transmitting power, bandwidth, and decoding complexity. However, multiple replicas of the signal can be transmitted using different techniques, such as by using different time slots (temporal diversity gain), different frequency slots (frequency diversity gain), and different polarization or transmitting antenna (spatial diversity gain). Temporal and frequency diversity gain techniques are not bandwidth efficient. Space-Time Codes (STC) [2,5,6] provides maximum diversity gain through spatial diversity, attaining better throughput, and coding gain but has a higher complexity. Space-Time Block Codes (STBC) [7], on the other hand, reduces the complexity, while at the same time providing diversity and coding gain. At the receiving end, the receiver either combines all of the copies of the signal to produce diversity gain or pick up the signal with the highest SNR among all the received signals. Therefore, two methods are used which is Maximum Ratio Combining (MRC) and Selection Combining (SC). More specifically, the MRC technique combines all of the signals received at one time for decoding the transmitted signal from N transmitting antennas and received at M receiving antennas, while providing a diversity gain of M times the SNR of the received signal. Consequently, the SC technique picks up the signal with the highest SNR having a diversity gain smaller as compared to the MRC technique, but with lower complexity, as it requires only one radio frequency chain. Both STC and STBC techniques use coherent detection, which required Channel State Information (CSI) or path gains matrix in the case of a spatial diversity in the fading channel [2]. Furthermore, CSI can be predicted by transmitting training symbols before transmitting the information, but, when the training sequences becomes outdated in fast fading, it directly increases the overhead and the power consumption.

In [8], the authors proposed a space-time signal construction method by combining orthogonal designs with sphere packings for the correlated Rayleigh fading channel that is inspired by space-time codes. The performance improvement of STBC scheme can be further improve by iteratively channel decoding along with the concatenation of sphere packing (SP) aided modulation [9]. Differential detection techniques are used to overcome the problem of finding the CSI. Therefore, multidimensional SP modulation assisted Differential Space Time Spreading assisted (SP-DSTS) provides better performance in terms of error probability and to overcome the channel dependency for decoding the received modulated symbols [10,11]. The proposed scheme uses the differential space time spreading (DSTS) technique, which encodes the symbol both in space domain by using two Tx antennas and in time domain by transmitting a symbol in two time periods of transmission for attaining diversity and coding gain. Moreover, DSTS is a suitable technique when the training symbols become outdated for each instance of a transmission. Differential is a complex scheme, but is reduces the computational complexity in fading channels.

Three different novel error protection schemes and two arrangements of partitioning order [A-B-C] and [B-A-C] are proposed. In partitioning order [A-B-C], the partitions types are prioritized as A, B, & C. Partition A is protected more than partition B, which is consequently protected more than partition C while using IRCC subcodes. Similarly, in partitioning order [B-A-C], the partitions are prioritized as B, A, and C, where partition B is protected more than partition A, which is protected more than partition C using the subcodes of multi-code IRCC. In multi-code IRCC, various constituent error protection subcodes are encoding the information bits according to their relative importance. The redundancy is iteratively utilized in the decoder side for the enhancement of high Bit Error Rate (BER) performance. A transmitter diversity gain technique is incorporated in order to further improve the performance of the proposed work, such that the coded video bitstream is passed to DSTS and SP modulation, which overcome channel estimation dependency. The motivation of this research work is to investigate the mechanism of performance improvement using iterative coding, while employing convergence capable inner and outer code, transmitter diversity gain using space-time signal construction, differential detection, and its impact on the performance of a highly compression efficient coded video stream. The motivations and contributions of the proposed work are summarized, as given below:

- Three different error protection schemes and two arrangements of H.264 partitions have been established.
- The proposed work uses irregular convolutional codes for seeking code-rates on which turbo cliff is obtained for achieving the prioritized partition.
- Space-time constellation has been included to observe the performance of the system on time-varying correlated Rayleigh fading channel.
- Space-time spreading is included, because it does not require the knowledge of the channel coefficients at the base.
- Space-time spreading codes only requires one spreading code per user.
- Space-time spreading method primarily targets systems that use two-transmitter and one-receiver antenna.
- Differential scheme has been incorporated in order to embed the information between successive symbols, in which we do not need to know the fade in estimation of the closest symbol.

The detailed discussion regarding previous work is carried out in Section 2. Section 3 presents preliminaries and system design criteria, while Section 4 presents the detailed description about the different parts of the proposed scheme. Section 5 presents the design of the IRCC. Section 6 provides a detailed explanation of the obtained performance results. Finally, the research article is concluded in Section 7.

## 2. Literature Review

There are two main problems in obtaining higher data-rates in a reliable communication, i.e., bandwidth limitation and channel fading. Shannon’s information-theoretic work [12] presented the basics of channel capacity bounds over a noisy communication channel and identified the most possible error-resilient features of communication systems. Channel codes are incorporated in the systems to effectively deal with the noisy channels. With bandwidth as a very precious commodity, maintaining a higher compression efficiency and data integrity is extremely important. Considerable factors affecting signal in wireless transmission are attenuation, shadowing, fading, and multi-user interference, which results in time and location-varying channel conditions. The first forward error correction block code for a single error correction was the Hamming code that was proposed in [13]. Elias was the first who discovered error-correcting convolutional codes in [14], in which the encoding dependencies shifted from finite-length segments to encoding dependencies that exist over the entire block. Turbo codes are first presented in [15], which comprises the concatenation of two Recursive Systematic Convoluational (RSC) codes attached together with an interleaver. In [16], Irregular-Variable length coding (IVLC) is presented, which endows its performance to near capacity on joint source channel coding.

The source coding part in Iterative Source Channel Decoding (ISCD) extracts the spectral coefficients from the multimedia source contents—i.e., audio or video signal. Residual redundancy remains in the coded coefficients after passing from the source codec in the form of non-uniform distribution and it can be manipulated at the receiver side to overcome the transmission errors. ISCD schemes operate on residual redundancy as a prime source for error protection in the coded video bitstream [17,18]. Moreover, for exploiting the performance of ISCD on residual redundancy in the source coded video bit-stream, a delightful Soft Bit Source Decoding (SBSD) scheme is proposed in [17]. Although, in the source coded bit-stream, very limited redundancy remains after the employment of the advanced compression techniques from the state-of-the-art compression standards. In [19], ISCD is proposed, which manipulates residual and artificial redundancy for minimizing the error robustness in digital systems, being motivated by concatenated codes. The authors proposed a convolutional neural network that was inspired by the advances in deep learning using iterative belief propagation architecture channel decoding under correlated noise [20]. Furthermore, a soft input decoder that is based on exploiting the residual redundancy in compressed bits and on the *A-Posteriori* probability of each symbol is presented. Moreover, the error-correcting or concealment capabilities of ISCD is evaluated by the EXtrinsic Information Transfer (EXIT) Chart.

In [21], the authors show the EXIT chart as a versatile tool for the design of different serial concatenation codes and a system design criteria on the EXIT chart has been briefly presented in [22]. The extent of performance achievement in terms of the convergence behavior of the iterative coding process in the form of the number of profitable iterations is determined by EXIT chart analysis in [23]. IrRegular Convolutions Codes (IRCCs) is proposed in [24], where the iterative decoding performance of the serially concatenated system is improved with the aid of EXIT chart using different constituent convolution codes having different code-rates.

With the potential to exploit the merits of source and channel decoding for mutual advantage, Joint Source Channel Decoding (JSCD) has received significant research interest in providing the lowest possible BER on realistic channel [19,25,26,27]. In [28], an analog low complexity Joint Source Channel Coding (JSCC) system is proposed for the transmission of still images. The authors in [29,30,31] evaluate the performance of the JSCC and find that it could be jointly optimized as one pair for achieving a lower BER. In [29], the authors proposed an algorithm, extended curve-fitting that finds optimal design criteria for JSCC. In [32], the authors uses and extended the application of Fully Parallel Turbo Decoder (FPTD) for the implementation of a unary error correction on hardware considering video transmission on JSCC [32]. Deep JSCC is proposed in [33] which does not explicitly relay on source and channel encoder and instead trained an auto-encoder composed of two convolutional neural network and can be used in a non-trainable layer in the middle of a communication channel. In [34], the authors proposed a lower complexity Robust Distributed Video Coding (RDVC) framework to optimize the quality of video communication for wireless multimedia sensors networks. A new coding scheme is presented based on Wyner-Ziv coding for error resilience and Rate Distortion (RD) performance. In [35], the authors proposed systematic code low density generator matrix (LDGM), which, under maximum likelihood decoding in terms of BER, can achieve the capacity of memoryless binary input output channel. The authors [36] proposed multi-edge type EXIT chart bit mapping for low density parity check Bit-interleaved Coded Modulation (BiCM).

In [37], the authors introduce the constellation-rotation technique to provide enterprise information security. Data security is provided over wireless transmission while using window-based fountain codes for edge computing. Specifically, H.264 video data are considered and the study has been carried by partitioning the video stream into windows ensuring latency into consideration while reducing the intercept probability. The authors in [38] studied rate adaptation technology of the MAC layer, application layer video codec technology, and packet encapsulation strategy of a transport layer. Moreover, the RTP packet encapsulation mode of H.264 data has been briefly studied and it implemented an adaptive packet encapsulation strategy based on channel quality estimation. In [39], the authors investigate energy optimized wireless video transmission using a hybrid automatic repeat request. The authors used H.264 as a source codec for maximizing the video quality subject to a constraint on the wireless transmission energy consumption.

The channel can be either blindly estimated or send training sequences before the transmission. Furthermore, the receiver cannot reliably decode the signal due to the destructive addition of multi-path propagation signals. The diversity technique is used, in which multiple replicas of the same signal are transmitted from multiple transmitting antennas. Differential schemes remove the estimation of CSI and, hence, reduce the overhead and complexity along with providing transmitting diversity gain. In fast fading channels, the training sequences becomes outdated very frequently, which linearly increases the overhead and energy consumption of the network. SP modulation was presented in [8] for transmission on correlated Rayleigh fading channel, while DSTS was presented in [10], which does not require the channel fade in estimating the signal. Table 1 illustrates a detailed comparative analysis of the previous work.

Against this background, the proposed novel arrangement of video transmission system invokes the service of Uni-code IRCC and the innate unequal error protecting capability of multi-code IRCC as an outer code, with the Unity Rate Coder (URC) as an inner code. Moreover, the proposed combination of inner and outer codes is capable of perfect convergence in iterative combination, while additional improvement is achieved using SP-DSTS [10,11]. The unequal error protection capability of IRCC ensures that the relatively important information bits undergo a very small number of channel errors by allocating more redundant bits to the relatively important bit-steam.

## 3. Preliminaries & System Design Criteria

The STBC technique requires channel estimation and uses coherent detection. The channel experiences an increase in the complexity and cost of the receiver due to the channel estimation technique. In comparison to this scheme, DSTS is constituted, which does not require any channel estimation technique. DSTS is a specific scheme for low computational complexity Multiple Input Multiple Output (MIMO) systems by using a non-coherent detection method. DSTS consists of a differential encoder and a space time spreading encoder (STS).

Space time spreading codes was originally designed as a transmitter diversity technique for the downlink of Code Division Multiple Access (CDMA) systems. A small number of Tx antennas at the base-station and one or more Rx antennas at the handset improved the performance by introducing a novel spreading codes while using the space-time codes. Constellation signals are spread in a balanced way to provide full diversity path gain at the receiver. The STS approach was designed to increase the bit rate and improve the quality and range in the downlink of either mobile or fixed CDMA systems [4].

**Figure 1:**
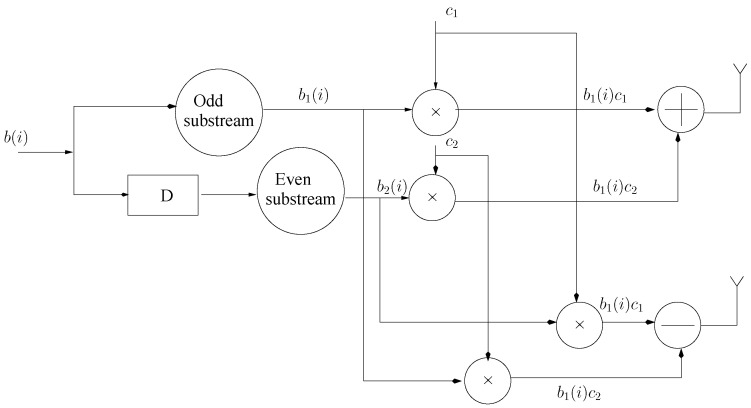
Space Time Spreading (STS) scheme.

The STS scheme divides the user data stream into two odd and even sub-streams, i.e., $b_{1}\left(i\right)$ and $b_{2}\left(i\right)$ refer to Figure 1 and produces the signals at both the transmitting antennas using orthogonal set of $c_{1}$ and $c_{2}$ unit norm spreading of a 2P×1 length, as given below:

(1) $$t_{1}=\frac{1}{\sqrt{2}}\left(b_{1}c_{1}+b_{2}c_{2}\right)$$

(2) $$t_{2}=\frac{1}{\sqrt{2}}\left(b_{2}c_{1}-b_{1}c_{2}\right)$$

**Figure 2:**
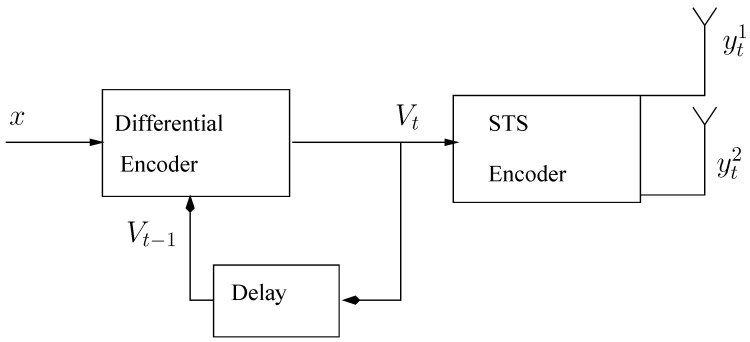
Differential Space Time Spreading (DSTS) Scheme.

The differential encoder take place before STS and then the encoded symbols are spread by using STS, where two symbols are transmitted while using two transmitter antennas. Initially, two dummy symbols $V_{0}^{1}$, $V_{0}^{2}$ are transmitted having no information and, afterwards, the bit-stream $x_{t}^{k};k=1,2$ at the bit-mapper are divided into $V_{t}^{1}$ and $V_{t}^{2}$ symbols refer to Figure 2. The differential encoded symbols are represented, as in [10,11].

(3) $$V_{t}^{1}=\frac{x_{t}^{1}\cdot V_{t-1}^{1}+x_{t}^{2}\cdot V_{t-1}^{2*}}{\sqrt{|V_{t-1}^{1}{|}^{2}+{\left|V_{t-1}^{2}\right|}^{2}}}$$

(4) $$V_{t}^{2}=\frac{x_{t}^{1}\cdot V_{t-1}^{2}-x_{t}^{2}\cdot V_{t-1}^{2*}}{\sqrt{|V_{t-1}^{1}{|}^{2}+{\left|V_{t-1}^{2}\right|}^{2}}}$$

Differential encoded symbols $V_{t}^{1}$ and $V_{t}^{2}$ are then spread into $\bar{C_{1}}$ and $\bar{C_{1}}$ using STS encoder. The consecutive differential encoded symbols at both transmitting antennas can be represented while using the following mapping [10,11].

(5) $$y_{t}^{1}=\frac{1}{\sqrt{2}}\left({\bar{C}}_{1}\cdot V_{t}^{1}+{\bar{C}}_{2}\cdot V_{t}^{2}\right)$$

(6) $$y_{t}^{2}=\frac{1}{\sqrt{2}}\left({\bar{C}}_{1}\cdot V_{t}^{1}-{\bar{C}}_{2}\cdot V_{t}^{2}\right)$$

In the case of N transmitting antenna, for the transmission purpose the average transmission power of each antenna $E_{s}=1/N$. The digital signal ($r_{t}^{j}$) at receiving antenna *j* after demodulation where $C_{t}^{j}$ represents the codeword and $\eta_{t}^{j}$ represents the path gain from the transmission antenna to the receiving antenna is given by the following equation [5].

(7) $$r_{t}^{j}=\sum_{i=1}^{N}\alpha_{i,j}C_{t}^{i}\sqrt{E}+\eta_{t}^{j}$$

The input and output relationships of Equation (7) are called the fading channel model, where α represents the channel path gain. The design criteria have been briefly studied for space time codes in [2], which guarantee maximum coding and diversity gain to improve the mutual information b/w the input and output of the system as a design criterion. For example, the $C_{1}$ codeword is selected at the transmission side from the codebook for transmission and, at the receiver, an error occurred if the decoder mistakenly estimates $C_{2}$.

(8) $$C^{2}=\left(\begin{array}{cccc} C_{1,1}^{2} & C_{1,2}^{2} & \cdots & C_{1,N}^{2}\\ C_{2,1}^{2} & C_{2,2}^{2} & \cdots & C_{2,N}^{2}\\ \vdots & \vdots & \ddots & \vdots \\ C_{T,1}^{2} & C_{T,2}^{2} & \cdots & C_{T,N}^{2} \end{array}\right)$$

If the codebook contains L number of codewords, then the probability of error using the union bound is given as:

(9) $$P\left(\;\mathrm{error}\;|C^{1}\;\mathrm{is}\;\mathrm{sent}\;\right)\leq \sum_{i=2}^{I}P\left(C^{1}\to C^{i}\right)$$

For an error matrix $D\left(C_{1},C_{2}\right)=C_{2}-C_{1}$, the pairwise error probability to be defined [2] in terms of non negative real number eigenvalues $\lambda_{n}\geq 0$ of matrix $A\left(C_{1},C_{2}\right)=D{\left(C_{1},C_{2}\right)}_{H}\cdot D\left(C_{1},C_{2}\right)$ $={\left(C_{2}-C_{1}\right)}_{H}\cdot \left(C_{2}-C_{1}\right)$, where *H* represents the conjugate transpose of the variable.

(10) $$P\left(C\to \mathrm{error}\right)\leq {\left(\prod_{n=1}^{r}\lambda_{n}\right)}^{-M}{\frac{E_{s}}{4N_{0}}}^{-rM}$$

or

(11) $$P\left(C^{1}\to C^{2}\right)\leq \frac{{\left(\frac{E_{s}}{4N_{0}}\right)}^{-rM}}{{\left(\prod_{n=1}^{r}\lambda_{n}\right)}^{M}}$$

or

(12) $$P\left(C^{1}\to C^{2}\right)\leq \frac{4^{rM}}{{\left(\prod_{n=1}^{r}\lambda_{n}\right)}^{M}\gamma^{rM}}$$

The diversity gain of the space time code is the rank of matrix $A(C_{1},C_{2})$ or the rank of the difference matrix $D(C_{1},C_{2})$ multiplied with the number of receiving antennas, which is the power of SNR in the denominator of Equation (12), i.e., rM, while the product of the nonzero eigenvalue matrix $A(C_{1},C_{2})$ corresponds to the coding gain. Consequently, a design criterion for a full diversity scheme is possible for M transmitting antennas and N receiving antennas if the matrix $A(C_{i},C_{i})$ is a full rank for all possible codewords and it is called a rank criterion. Contrary, the coding gain is the distance between two codes and it is equal to $det(A\left(C_{1},C_{2}\right))$. The coding gain can be increased by maximizing the minimum determinate of matrix $A(C_{1},C_{2})$, which is the determinant criterion [2]. Because the symbols in STBC are always orthogonal, the two orthogonal symbols transmitted from 2 Tx antennas at $t_{1}$ and $t_{2}$ can be represented with $C_{1}=\left[\begin{array}{cc} x_{1} & x_{2}\\ -x_{2}^{*} & x_{1}^{*} \end{array}\right]$. The first Tx antenna transmit the original signal while the second transmit the conjugate version of the original signal. For instance, at the receiver, the following pair of symbols received: $C_{2}=\left[\begin{array}{cc} x_{1}^{{}^{\prime }} & x_{2}^{{}^{\prime }}\\ -x_{2}^{{}^{\prime }*} & x_{1}^{{}^{\prime }*} \end{array}\right]$. The difference metric can be expressed as: $D\left(C_{1},C_{2}\right)=\left[\begin{array}{cc} x_{1}^{{}^{\prime }}-x_{1} & x_{2}^{{}^{\prime }}-x_{2}\\ x_{2}^{*}-x_{2}^{{}^{\prime }*} & x_{1}^{{}^{\prime }*}-x_{1}^{*} \end{array}\right]$. The determinant of the difference matrix $det\left[C_{1},C_{2}\right]={\left[x_{1}^{{}^{\prime }}-x_{1}\right]}^{2}+{\left[x_{2}^{{}^{\prime }}-x_{2}\right]}^{2}$ is equal to 2 if $C_{1}\neq C_{2}$, otherwise it is zero, which shows that the difference matrix is a full rank for Alamouti code and it satisfies the determinant criteria.

Therefore, STBC has to be a good code, if (a) the rank of the difference matrix b/w any two codewords is a full rank and (b) if there exists a fast maximum likelihood decoding algorithm. The space time block codes from orthogonal designs guarantee maximum diversity and fast maximum likelihood designs because of the special structure of orthogonal designs [41]. SP modulation was designed for obtaining full diversity in the time-correlated Rayleigh fading channel by combining orthogonal designs with sphere packing inspired by space-time codes [8]. A recursive orthogonal design function can be constructed, as given below [8,41]. Let $G1\left(x\right)=x_{1}I_{1}$, where $I_{1}$ is the identity matrix.

(13) $$G_{2^{k}}\left(x_{1},\cdots ,x_{k+1}\right)=\left[\begin{array}{cc} G_{2^{k-1}}\left(x_{1},\cdots ,x_{k}\right) & x_{k+1}I_{2^{k-1}}\\ -x_{k+1}^{*}I_{2^{k-1}} & G_{2^{k-1}}^{H}\left(x_{1},\cdots ,x_{k}\right) \end{array}\right]$$

with $x_{1},x_{2},.....x_{k+1}$ are complex variables and $x_{1}^{*},x_{2}^{*},.....x_{k+1}^{*}$ are their conjugate version. Similarly, H denotes the transpose and conjugate of $G_{2^{k-1}}$. A set of SP signals can be constructed from orthogonal design of $G_{2^{k}}$, as given below.

(14) $$C=\sqrt{2^{k}/\left(k+1\right)}G_{2^{k}}\left(x_{1},x_{2},\cdots ,x_{k+1}\right)$$

Here, $\sqrt{2^{k}/\left(k+1\right)}$ is the normalization factor for energy constraint and $k+1/2^{k}$ is the symbol rate. For instance, the SP signals for two Tx antennas are given below by exploring the above equation.

(15) $$C=\sqrt{2^{k}/\left(k+1\right)}G_{2}\left(x_{1},x_{2}\right)=\sqrt{2^{k}/\left(k+1\right)}\left[\begin{array}{cc} G_{1}\left(x_{1}\right) & x_{2}\\ -x_{2}^{*} & G_{1}^{H}\left(x_{1}\right) \end{array}\right]=\sqrt{2^{k}/\left(k+1\right)}\left[\begin{array}{cc} x_{1} & x_{2}\\ -x_{2}^{*} & x_{1}^{*} \end{array}\right]$$

Similarly, for four and eight Tx antennas, the SP signals can be obtained as:

(16) $$C=\sqrt{2^{k}/\left(k+1\right)}G_{4}\left(x_{1},x_{2},x_{3}\right)=\sqrt{2^{k}/\left(k+1\right)}\left[\begin{array}{cc} G_{2}\left(x_{1},x_{2}\right) & x_{3}I_{2}\\ -x_{3}^{*}I_{2} & G_{2}^{H}\left(x_{1},x_{2}\right) \end{array}\right]$$

(17) $$=\sqrt{2^{k}/\left(k+1\right)}\left[\begin{array}{cccc} x_{1} & x_{2} & x_{3} & 0\\ -x_{2}^{*} & x_{1}^{*} & 0 & x_{3}\\ -x_{3}^{*} & 0 & x_{1}^{*} & -x_{2}\\ 0 & -x_{3}^{*} & x_{2}^{*} & x_{1} \end{array}\right]$$

(18) $$C=\sqrt{2^{k}/\left(k+1\right)}G_{8}\left(x_{1},x_{2},x_{3},x_{4}\right)=\sqrt{2^{k}/\left(k+1\right)}\left[\begin{array}{cc} G_{4}\left(x_{1},x_{2},x_{3}\right) & x_{4}I_{4}\\ -x_{4}^{*}I_{4} & G_{4}^{H}\left(x_{1},x_{2},x_{3}\right) \end{array}\right]$$

(19) $$=\sqrt{2^{k}/\left(k+1\right)}\left[\begin{array}{cccccccc} x_{1} & x_{2} & x_{3} & 0 & x_{4} & 0 & 0 & 0\\ -x_{2}^{*} & x_{1}^{*} & 0 & x_{3} & 0 & x_{4} & 0 & 0\\ -x_{3}^{*} & 0 & x_{1}^{*} & -x_{2} & 0 & 0 & x_{4} & 0\\ 0 & -x_{3}^{*} & x_{2}^{*} & x_{1} & 0 & 0 & 0 & x_{4}\\ -x_{4}^{*} & 0 & 0 & 0 & x_{1}^{*} & -x_{2} & -x_{3} & 0\\ 0 & -x_{4}^{*} & 0 & 0 & x_{2}^{*} & x_{1} & 0 & -x_{3}\\ 0 & 0 & -x_{4}^{*} & 0 & x_{3}^{*} & 0 & x_{1} & x_{2}\\ 0 & 0 & 0 & -x_{4}^{*} & 0 & x_{3}^{*} & -x_{2}^{*} & x_{1}^{*} \end{array}\right].$$

## 4. Proposed System Setup

**Figure 3:**
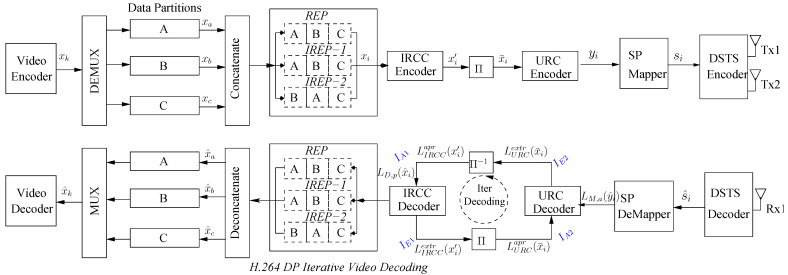
Proposed System Model.

A single bit in error or burst of errors in the received bitstream may influence the correct decoding of several succeeding frames in the predictive coding paradigm. Therefore, a clever system has to be designed to ensure an acceptable video quality in limiting the channel errors to a minimum acceptable level. This work proposes a complex video phone arrangement for the transmission of video bit-stream on a correlated Rayleigh fading channel, as shown in Figure 3. The different fragments of the proposed video phone arrangements are explained in the following subsections.

### 4.1. Source Encoder H.264 Compression Standard

H.264 Advanced Video Coding Standard (AVC) [42] is the advance compression standard that has gained popularity due to its well acceptable nature in different diverse application scenarios and the provision of greater compression efficiency with flexibility to operate in different network conditions [43]. H.264/AVC provides a network-friendly representation for both conversational—e.g., video telephony, multimedia messaging, and non-conversational—e.g., broadcast, storage, distribution of video contents, video on demand, and multimedia streaming applications. The design of H.264/AVC video codec aimed to enable the interoperability between the encoder and decoder of different manufactures while achieving the high coding efficiency [43]. The H.264/AVC encodes the input video stream into two layers—i.e., Video Coding Layer (VCL) and Network Adaptation Layer (NAL). Moreover, VCL represents the coded bit-stream, while NAL ensured the compatibility of the coded bit-stream in various types of networks. The coded frame is organized into slices, where each slice comprises Macroblocks (MBs), and each MB is categorized into Intra-coded and Inter-coded types. More specifically, the MB in Inter-coded mode is transmitted with the motion vector, and the displacement residual error is calculated at the receiver side in order to reconstruct the original MB, while, in Intra-coded mode, the MBs are encoded without any reference to previously transmitted information MBs. Furthermore, the error-resilient Data Partitioning (DP) technique allows for the prioritized manipulation of the coded stream. Three different partitions, comprising of type A, B, and C, exist per frame in the DP mode. The priority has been given to each according to their importance and type. Type A partition contains very important information, including coded header information, a motion vector, slice header, quantization parameters, and MB types. Type B partition is also an important partition containing Intra Coded Block Pattern (CBP) bits and MB coefficient. Similarly, partition C contains inter-frame motion-compensated residual error bits for MB encoded by inter-frame prediction. Furthermore, it is important to note that, if, in case the type A partition is corrupted, then the whole block is considered distorted and marked as corrupted and, consequently, the decoder applies error concealment technique to compensate the impact of errors. Upon the presence of partition A and B, an intra-frame MB update per frame is added in the reconstructed frame, while motion residual bits are consequently incorporated if type C partition is received without any errors induced by the channel noise.

### 4.2. Source Coding Arrangements

The raw video captured at the source encoder is compressed at the transmitter side using the H.264/AVC compression standard. The video encoder encodes the input video into a stream of $x_{k}$ or *k* information bits. Consequently, the coded stream is then processed by the DEMUX block, which further segregates the encoded stream into their respective type A, B, and C data partitions that are represented as $x_{a}$, $x_{b}$, and $x_{c}$, which are further concatenated in different orders, depending on the applied error protection schemes, as in Table 2 and Table 3. Thereafter, the concatenated bitstream $x_{i}$ is presented to the channel encoder for further processing.

### 4.3. Channel Coding Arrangements

IRCC outer code is used in combination with URC as an inner channel encoder. Channel codes operates for the removal of the channel errors. The output codeword $x_{i}^{{}^{\prime }}$ from a set of a codeword of IRCC encoder is interleaved (Π) for protecting the bits from a burst of errors by randomizing the bitstream through permutation. Interleaved sequence ($\bar{x_{i}}$) is further presented to URC. The overall code rate of the system is $R=IRCC_{code-rate}\cdot URC_{code-rate}$. The processed output $y_{i}$ of the URC is presented to the transmission system for its delivery to the receiver.

### 4.4. SP Modulation Aided DSTS Based Transmission Arrangements

The output $y_{i}$ of the inner URC code is fed as an input to the SP mapper. SP modulation is used to keep the maximum possible euclidean distance between the modulated symbols. Thereafter, the stream is then modulated into constellation symbols $S_{i}$, where i=1,2,3,⋯L−1 having *L* modulated signal alphabet. The modulated symbols are then further transmitted by DSTS using two transmitter antennas.

### 4.5. Iterative Source Channel Decoding Setup:

The input alphabet is referred to the set of elementary input signals and the individual signals as an input symbols. Consequently, the output alphabet is a set of regions in channel output space and the individual signal as an output symbols. The channel output symbol is generated by the following expression, provided that the bits are independent of each other [17,44].

(20) $$P\left[{\hat{y}}_{\tau }|y_{\tau }\right]=\prod_{k=1}^{K}P\left[{\hat{y}}_{\tau }\left(k\right)|y_{\tau }\left(k\right)\right]$$

where ${\hat{y}}_{\tau }$ is the received information on the transmission of $y_{\tau }.$ For a specific bit $y_{\tau }\left(\lambda \right),$ the output extrinsic information $P\left[{\hat{y}}_{\tau }^{\left[ext\right]}|y_{\tau }^{\left[ext\right]}\right]$ can be achieved as follow.

(21) $$P\left[{\hat{y}}_{\tau }^{\left[ext\right]}|y_{\tau }^{\left[ext\right]}\right]=\prod_{k=1,k\neq \lambda }^{K}P\left[{\hat{y}}_{\tau }\left(k\right)|y_{\tau }\left(k\right)\right]$$

Eventually, the channel output information is combined with the A-priori knowledge of that corresponding symbol in order to achieve the resultant Log-Likelihood Ratio (LLR) extrinsic value, as expressed below.

(22) $$\begin{array}{c} \mathrm{LLR}\left[y_{\tau }\left(\lambda \right)\right]=\\ \log\left(\frac{\sum_{y_{\tau }^{\left[ext\right]}}P\left[y_{\tau }^{\left[ext\right]}|y_{\tau }\left(\lambda \right)=+1\right]\cdot P\left[{\hat{y}}_{\tau }^{\left[ext\right]}|y_{\tau }^{\left[ext\right]}\right]}{\sum_{y_{\tau }^{\left[ext\right]}P}\left[y_{\tau }^{\left[ext\right]}|y_{\tau }\left(\lambda \right)=-1\right]\cdot P\left[{\hat{y}}_{\tau }^{\left[ext\right]}|y_{\tau }^{\left[ext\right]}\right]}\right) \end{array}$$

Mutual information or A-priori knowledge is a measure of information that one random variable has about the others [45]. Entropy becomes the self-information of a random variable. Mutual information is a more general quantity, called a relative entropy, a measure of the distance between two probability distributions. Symbol-wise mutual information between symbol $S_{i}$ and received Rayleigh fading channel output ${\hat{y}}_{\tau }$ is given by the following equation [21]:

(23) $$I\left(S_{i};{\hat{y}}_{\tau }\right)=\frac{1}{L}\cdot \sum_{n=1}^{L}\int_{-\infty }^{+\infty }p\left({\hat{y}}_{\tau }|S_{i}=a_{n}\right)\times ld\frac{p\left({\hat{y}}_{\tau }|S_{i}=a_{n}\right)}{p\left({\hat{y}}_{\tau }\right)}dz$$

where $a_{n}$ represents the current signaling element. The conditional Probability Density Function (PDF) of Equation (23) is given as:

(24) $$p\left({\hat{y}}_{\tau }|S_{i}=a_{n}\right)=\frac{1}{\sqrt{2\pi }\sigma }\cdot \exp\left[\frac{\left[-{\left({\hat{y}}_{\tau }-an\right)}^{2}\right]}{2\sigma^{2}}\right]$$

where

(25) $$p\left({\hat{y}}_{\tau }\right)=\frac{1}{L}\sum_{n=1}^{L}p\left({\hat{y}}_{\tau }|S_{i}=a_{n}\right)$$

At the receiver side in the iterative decoding paradigm, the constellation signal is iteratively decoded by exchanging the mutual information between the inner and outer decoder, which iteratively collaborate in order to give the highest possible output to the source decoder. Furthermore, the received symbols are first presented to the DSTS de-mapper and are further demodulated into $L_{M,a}\left(\hat{y_{i}}\right)$ by SP demodulation as mentioned in Figure 3. At first iteration only the LLR values $L_{M,a}\left(\hat{y_{i}}\right)$ are presented to the URC decoder, but, in the consequent iterations, both the LLR values $L_{M,a}\left(\hat{y_{i}}\right)$ and *A*—priori information $L_{URC}^{apr}$ are presented to the URC decoder. *A*-posteriori information $L_{M,a}\left(\hat{y_{i}}\right)$ is then subtracted from *A*-priori information $L_{URC}^{apr}\left(\bar{x_{i}}\right)$ to obtain the extrinsic information $L_{URC}^{extr}\left(\bar{x_{i}}\right)$, which is further deinterleaved and presented as input *A*-priori soft bit sequence $L_{\mathrm{IRCC}\;}^{\mathrm{apr}(x_{i}^{{}^{\prime }})}$ to the IRCC decoder, which, in turn, uses the Maximum A-posteriori Probability (MAP) decoder for decoding. The output *A* -posteriori LLR $\left(L_{D,p}\right)$ of MAP decoder is subtracted from the soft bit sequence ($L_{IRCC}^{\mathrm{apr}}$), which results in the extrinsic information $L_{IRCC}^{extr}$. The extrinsic information is further interleaved to become the *A*-priori information ($L_{URC}^{\mathrm{apr}}$) for the next iteration. Furthermore, the performance of IRCC and URC completely depends on the number of iterations of the iterative process and it completely depends on residual redundancy that remains in the coded bit-stream.

### 4.6. Source Decoding and Video Quality Evaluation:

Afterwards, the profitable number of iterations of iterative decoding comes to an end and the round is off; the soft-bit output sequence ($L_{D,p}\left({\hat{x}}_{1}\right)$) of the correspondence scheme is deconcatenated into type $A\left({\hat{x}}_{a}\right),$ type $B\left({\hat{x}}_{b}\right),$ and type $C\left({\hat{x}}_{c}\right)$ partitions and is presented to the MUX block. The MUX block converted the partitions (A,B, and C) into their resultant output stream of ${\hat{x}}_{k}$, which is finally presented to the employed AVC decoder in order to generate the final decoded video sequence.

## 5. IRCCs Channel Code Design

IRCC comprising of $I_{n}$ constituent subcodes that are constructed by selecting the basic main code having code-rate—$r_{1}$ and the rest of the subcodes ($I_{n}-1$) are designed in correspondence with that main code [24,46]. The convergence behavior of iterative decoding is improved by designing irregular codes that are based on a simplified efficient optimization algorithm. Convergence is sometimes possible through specific code-rates on scanning the proper code through many possible subcodes. The search procedure can be reduced by defining a family of subcodes and it is generally referred to as the irregular code [24]. The subcodes ([$I_{n}-1$]) in which the codewords having $rate-r_{i}>r_{1}$ (higher rates than the main code), where i=1,2,3…$I_{n}$ is achieved by puncturing and the codewords of rate—$r_{i}<r_{1}$ (lower rates than the main code) is obtained by adding more generator polynomials. The encoder of IRCC codes encodes *k* information bits into *n* coded bits, and each subcode encodes a fraction $\left(\alpha_{i}r_{i}n\right)$ of information bits and generated $\left(\alpha_{i}n\right)$ coded bits. Furthermore, the weighting coefficient ($\alpha_{i}$) represents the relative size of a fraction or weighting coefficient with $i=1,2,3\ldots .I_{n}$, while the target code-rate Rε[01] should satisfy the following condition. $\sum_{i=1}^{l_{n}}\alpha_{i}=1$ and

$$\sum_{i=1}^{l_{n}}\alpha_{i}r_{i}=R,\;\;\mathrm{where}\;\alpha_{i}\in \left[0\;1\right]$$

As a design example, the iterative paradigm of the inner and outer codes operates at a constant overall bit budget rate of 3/4 and, logically, the redundancy is incorporated by the outer IRCC code and the inner code is operated at the unity rate. IRCC uni-code is used as a reference scheme, which employs the rate 3/4 single constituent code for regular channel coding of the H. 264 partitions, while a subset of the 17 constituent subcodes of the considered IRCC is utilized using the optimization procedure that was defined in [24], in order to perform irregular error protection of the H.264/AVC coded video. The mutual information $\left(I_{A1}\right)$ for outer IRCC decoder is the information sharing between the A-priori information $\left(L_{IRCC}^{\mathrm{apr}}\left(x_{i}^{\prime }\right)\right)$ and the transmitted information $\left(L_{D,p}\left({\hat{x}}_{i}\right)\right)$, as given below.

(26) $$I_{A1}=I\left(L_{D,p}\left({\hat{x}}_{i}\right);L_{IRCC}^{apr}\left(x_{i}^{\prime }\right)\right)$$

Mutual information $\left(I_{E1}\right)$ is the information transfer between the extrinsic information $\left(L_{IRCC}^{\mathrm{extr}\;}\left(x_{i}^{\prime }\right)\right)$ and transmitted information $\left(L_{D,p}\right)$ i.e.,

(27) $$I_{E1}=I\left(L_{D,p}\left({\hat{x}}_{i}\right);L_{IRCC}^{\mathrm{extr}\;}\left(x_{i}^{\prime }\right)\right))$$

The outer IRCC convergence characteristic transfer function can also be obtained, as given below:

(28) $$I_{E1}=T_{IRCC-\mathrm{code}}\left(I_{A1}\right)$$

Similarly, the mutual information $\left(I_{A2}\right)$ for URC decoder is the information sharing between the A-priori information $\left(L_{URC}^{apr}\left({\bar{x}}_{i}\right)\right)$ and the transmitted information $\left(L_{M,a}\left(\hat{y}\right)\right)$ i.e.,

(29) $$I_{A2}=I\left(L_{D,p}\left({\hat{x}}_{i}\right);L_{URC}^{apr}\left({\bar{x}}_{i}\right)\right)$$

While

(30) $$I_{E2}=I\left(L_{D,p}\left({\hat{x}}_{i}\right);L_{URC}^{etr}\left({\bar{x}}_{i}\right)\right)$$

and the transfer characteristic function $\left(I_{E2}\right)$ for Rate- 1 precoder is dependent on the $E_{b}/N_{0}$ received from the channel and as well in the mapping between $I_{A2}$ and $I_{E2}$, as denoted by the following equation.

(31) $$I_{E2}=T_{URC-Code}\left(I_{A2},E_{b}/N_{0}\right)$$

The resultant output information after *k* iterations at the outer decoder can be obtained from the following equation [46].

(32) $$\Omega_{k}=T_{IRCC}\left(T_{URC}\left(\Omega_{k-1},E_{b}/N_{0}\right)\right)$$

**Figure 4:**
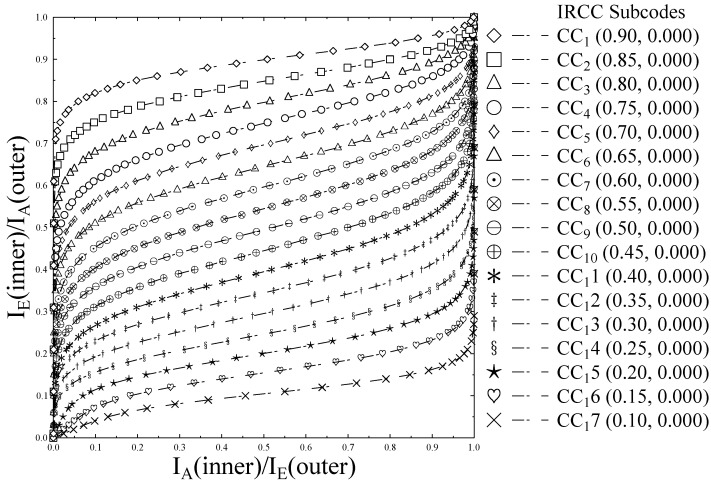
EXtrinsic Information Transfer (EXIT) characteristic curves of IRCC 17 constituent subcodes.

In our considered irregular error protection mechanism, we utilized IRCC having subcode-rates $r_{i}$ = [0.10, 0.15, 0.20, 0.25, 0.30, 0.35, 0.40, …0.90]. Therefore, the employed IRCC is capable of providing different error protections to the coded bitstream and it can be varied, depending on the specific order of concatenation of the three H.264/AVC partitions per video frame. The IRCC percentage vector derived using the IRCC optimization procedure reveals a IRCC subcode *r* with code-rate =0.30 encode 0.6% of the stream, while that of other subcodes encodes the corresponding portion of the video stream according to the input percentage vector. Figure 4 shows the EXIT chart of the 17 subcodes of IRCC. It is justified from the figure that different code-rates produces different curves on the EXIT chart, used to find the intersection with the inner precoder curve, at which turbo cliff is achieved.

## 6. Results and Discussion

The proposed system is designed with a motivation to provide a various error protection scheme based on data partitioning technique of H.264 while utilizing the innate unequal error protection capability of the employed channel codes. Furthermore, space-time signal construction or SP modulation aided DSTS technique has been incorporated to provide the video quality to mobile handset systems on time-varying correlated Rayleigh fading.

### 6.1. EXIT Chart Analysis of the Constituent Decoders

ISCD schemes operate on residual redundancy as a prime source of error protection in the coded video bitstream. More specifically, the decoder exchanges the information b/w the constituent decoders to obtain the highest possible information regarding the transmitted stream.

The main goal of iterative channel decoding is to glean the highest possible extrinsic information $\left[L_{IRCC}^{\mathrm{extr}\;}\left(x_{i}\right)\wedge L_{\mathrm{URC}\;}^{\mathrm{extr}\;}\left(x_{i}\right)\right]$. The constituent inner and outer decoder in iterative channel decoding iteratively assist each other to extract the maximum possible extrinsic information. The idea of the EXIT chart is presented by Stephen ten Brink in order to examine the exchange of mutual information between the constituent inner and outer decoders, employed in an iterative decoding fashion [47]. The detailed description regarding A-priori information ($I_{A}$), A-posteriori LLR($I_{E}$), and Extrinsic Information ($I_{E}$) can be found in [30].

**Figure 5:**
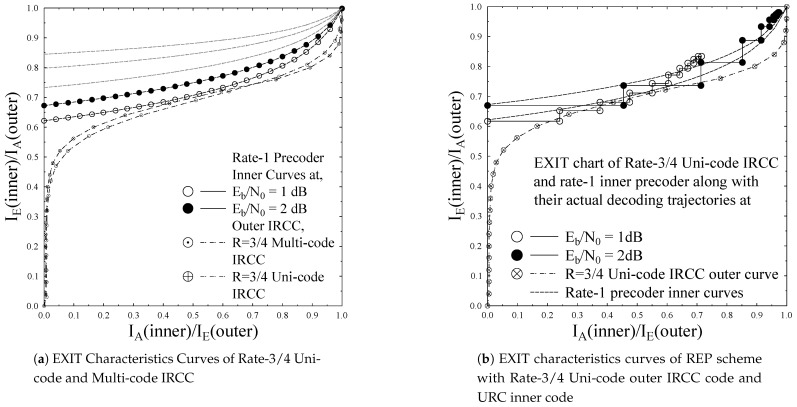

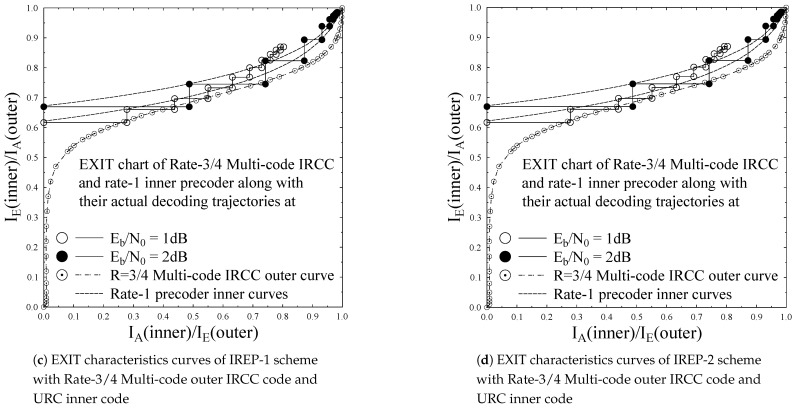
EXIT curves of the three different error protected schemes.

The results that are presented in the paper were obtained by employing Monte Carlo simulation model. The simulations were carried out using 45 frames of the “AKIYO” video sequence as input vide stream, while experiments were repeated for 260 times for the considered transmission conditions and its average considered as the expected outcome. The amount of available a-priori knowledge at the demapper dictates the extrinsic information transfer. EXIT chart is plotted between demapper and decoder for observing the exchange of extrinsic information. The channel inner extrinsic information output $I_{E1}$ becomes the outer a-priori input $I_{A2}$, while the outer extrinsic information output $I_{E2}$ becomes the inner a-priori knowledge $I_{A1}$, as plotted on the y-axis and x-axis, correspondingly. EXIT chart predictions are verified by the simulation results from the decoding trajectories. The decoding transfer characteristic is plotted for both IRCC unicode and IRCC multicode with precoder at $E_{b}/N_{0}$ = 1 dB, and 2 dB, as shown in Figure 5a. The IRCC unicode intersect at lower mutual information, while the IRCC multicode intersects at higher mutual information, which shows that the IRCC multicode results in a wider open tunnel between the demapper and decoder. From Figure 5b, the decoding transfer intersect and decoding trajectory of the IRCC unicode is stuck after five iterations at lower mutual information, which results in a higher BER rate at $E_{b}/N_{0}$ = 1 dB, while, for the same IRCC unicode, the decoding trajectory intersects at slightly higher mutual information, but does not achieve the perfect convergence, such as $\left(I_{A},I_{E}\right)=\left(1,1\right)$ at $E_{b}/N_{0}$ = 2 dB. Similarly, Figure 5c,d show the decoding trajectory for IREP-1 and IREP-2, correspondingly. For $E_{b}/N_{0}$ of 1 dB, the decoding transfer has limited performance after five iterations, while, for $E_{b}/N_{0}$ = 2 dB, due to an open EXIT tunnel, the performance gain is high, due to iterative convergence close to $\left(I_{E},I_{A}\right)=\left(1,1\right)$ point. Figure 5a records and depicts the outer EXIT characteristic curves of the IRCC multicode and the IRCC unicode. The analysis shows that the IRCC multicode results in a relatively wider open tunnel in combination with the inner URC code as compared to that of the counterpart with the same 3/4 code-rate. Furthermore, the IRCC multicode has the innate irregular capability to irregularly treat the H.264/AVC coded stream having three different types of partitions with varying relative importance. Therefore, our system design with a IRCC multicode for a similar code rate as that of unicode would have the additional advantage of irregular treatment capability for the H.264/AVC coded stream. More specifically, the EXIT charts for REP, IREP-1, and IREP-2 schemes of Table 3, along with their actual decoding trajectories at $E_{b}/N_{0}$ values of 1 dB and 2 dB, with a consistent overall code rate of 3/4, are plotted in Figure 5b–d. It can be observed from the decoding trajectories of Figure 5b–d that the IREP schemes have relatively better convergence behavior when compared to the REP scheme. This improvement is manifested, due to the provision of a relatively wider EXIT tunnel for the IREP scheme. Furthermore, it can be observed from the EXIT curves of Figure 5c,d that the employed IREP-1 and IREP-2 have similar EXIT characteristic curves and decoding trajectories at $E_{b}/N_{0}$ value of 1 dB and 2 dB, because the IREP-1 and IREP-2 have employed the same inner and outer codes, and the only difference is in the order of presentation of the coded stream to the iterative coding-decoding setup.

### 6.2. Subjective and Objective Performance Analysis

Subjective and objective techniques are widely used for measuring the video quality. Human participants are required in the subjective technique to assess the streaming quality and, therefore, it is a time-consuming solution. On the other hand, different methods are used in objective measures, such as BER and Peak Signal to Noise Ratio (PSNR). PSNR is a valid metric for measuring the video quality when the video contents and codecs remain unchanged [48]. For simulation purposes, the “AKIYO” video sequence, having extremely low dynamism and having fine details in the background, is utilized and encoded using H.264/AVC JM 19 video codec. Because of the low dynamism of the video sequence and the considered low-bit-rate videophone scenario, the Flexible Macroblock Ordering (FMO) error-resilient technique is turned off to reduce the computational complexity of the considered real-time video communication system scenario. Furthermore, the considered video sequence consists of 45 frames having a QCIF (176 × 144) pixel format with a temporal resolution of 15 frames per second (fps). The arrangement of the Intra-coded I-frame is one out of 44 predicted P-frames. In order to limit the error propagation within the frame boundaries, each frame is subdivided into nine slices, while each slice consisting of 11 macroblocks. Moreover, only one reference frame is used in the inter-frame prediction instead of multiple reference frames in order to keep lower the complexity of the employed video coding setup. The number of profitable iterations is set to seven in the iterative decoding operation. Monte Carlo simulations were carried out using 45 frames of the “AKIYO” video sequence and the experiments were repeated 260 times and the average results are considered.

The performance of the three different error protection schemes, which include REP scheme, Irregular Error Protection scheme-1 (IREP-1), and Irregular Error Protection scheme-2 (IREP-2) listed in table-II, are evaluated and compared. The REP scheme uses Uni-code IRCC by only employing the rate-3/4 subcode of the constituent IRCC code for all three partitions, while for IREP-1 and IREP-2, a subset of the 17 subcodes of the employed IRCC is utilized. Using Multi-code IRCC, the source coded streams are irregular error protected, depending on the code-rate of the constituent sub-code. In IREP-1, the concatenation order of [A-B-C] is followed. As a result, in IREP-1, type A partition is provided with the highest error protection as compared to both type B and C partitions, while the type B partition has high error protection than partition C. Similarly, the concatenation order [B-A-C] is considered in IREP-2. In the concatenation order [B-A-C], type B partition has higher protection when compared to the other two partitions i.e., type A and type C and type A is protected more than type C partition.

**Figure 6:**
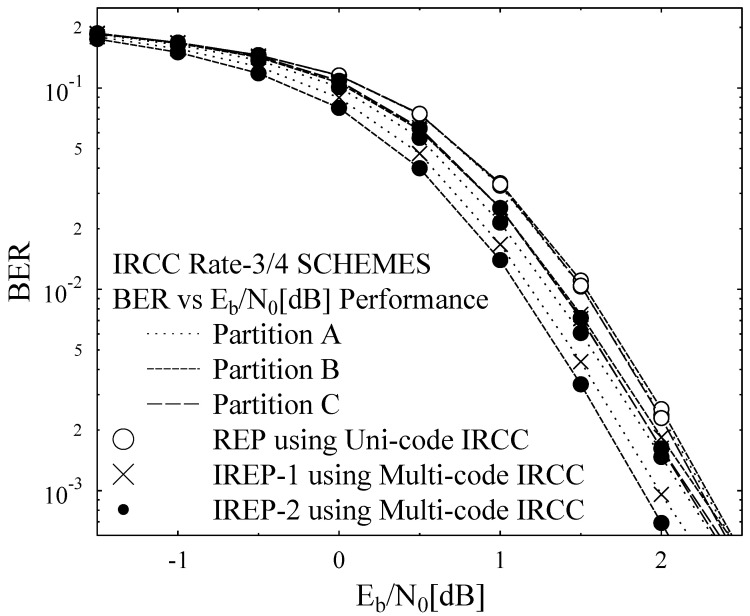
Bit Error Rate (BER) Performance Curves of the proposed schemes with IREP-2 employing error protection order[B-A-C].

**Figure 7:**
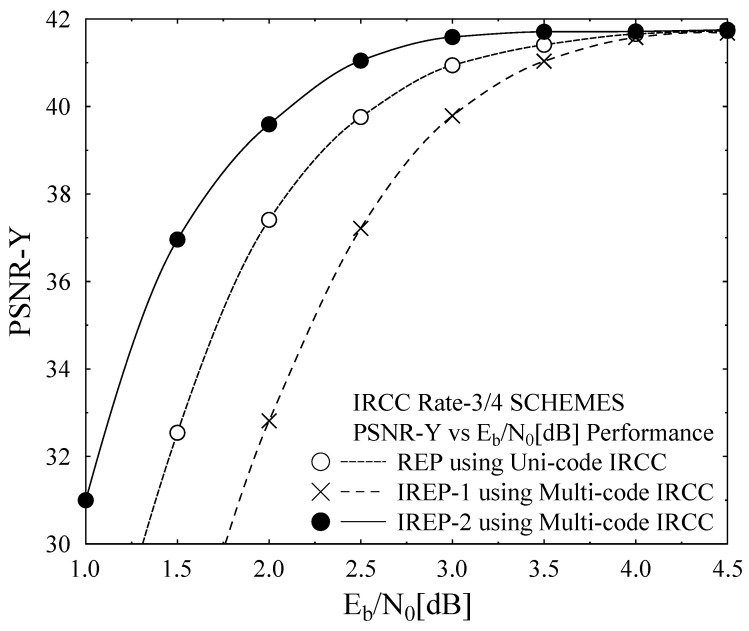
Peak Signal to Noise Ratio (PSNR) Performance Curves of the Coding Scheme.

**Figure 8:**
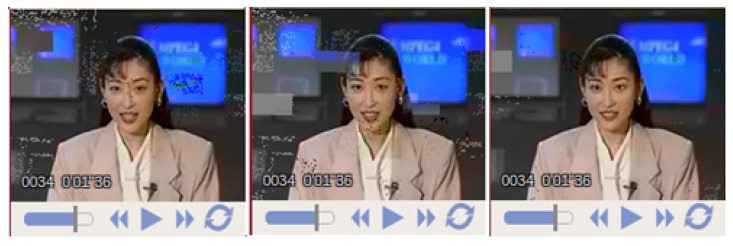
Subjective video quality of the 34th frame of AKIYO video sequence for the employed REP, IREP-1, and IREP-2 schemes (from left to right).

Figure 6 presents the BER performance of the employed error protection schemes, employing IRCC and URC in iterative channel decoding fashion and while using SP modulation aided DSTS mechanism for the transmission of H.264/AVC coded “AKIYO” video sequence. In Figure 6, the BER for all three schemes are plotted with varying $E_{b}/N_{0}$ value and maintaining the overall bit-rate consistent, while transmitting the considered “AKIYO” video sequence. REP performs the same for all three partitions of type A, B, and C, due to the employment of the same Uni-code IRCC for all three types of partitions which has given the same level of protection to type A, B, and C partitions, as presented in Figure 6. More specifically, the priority of protection is higher for the type A partition in the IREP-1 scheme with the help of applying lower constituent code-rates subcodes when compared to partition type B and C of the same scheme, as can be confirmed from the BER performance curves that are shown in Figure 6. Similarly, for the IREP-2 scheme, the type B partition has better BER performance when compared to type A partition and type C partition, while partition C has a higher BER relative to partition A and it can be observed from the BER performance curves that are presented in Figure 6. The closer approximation of the EXIT chart demonstrates that a pinch-off achieved a lower $E_{b}/N_{0}$ of 2dB for IREP-1 and IREP-2 where the curves just intersect at less iterations and, consequently, results in a lower BER. At the right side of the EXIT chart, the intersection between the demapper and decoder transfer chart dominates the BER floor. It can be observed from the BER performance curves of Figure 6 that the IREP-1 and IREP-2 schemes have relatively better BER performance when compared to the REP scheme. This performance improvement for the IREP-1 and IREP-2 is owed to the provision of a wider open tunnel for the constituent inner and outer codes of the IREP-1 and IREP-2 schemes. In [30], the convergence behaviors of SECCCs is analyzed with respect to the benchmark systems, while considering Single Input Single Output (SISO) transmission over Rayleigh channel. With reference to [30], our proposed system employs Irregular Convolutional Codes as a channel codes along with space-time codes for providing the diversity gain in the Multiple Input Multiple Output (MIMO) transmission mechanism. The focus of the proposed work is to achieve the performance gain by exploiting the irregular error protection capability of Irregular Convolutional Codes as channel codes and DSTS-SP for providing diversity gain and independence in the fading channel. With reference to [30], our proposed IREP-2 achieved a performance gain of 1.5 dB at the BER degradation point of 10-1. Figure 7 presents the PSNR Vs $E_{b}/N_{0}$ performance curves of the proposed REP, IREP-1, and IREP-2 schemes. It can be observed in Figure 7 that IREP-1 provides the worst PSNR performance, while IREP-2 provides the best PSNR performance as compared to IREP-1 and the REP schemes. More specifically, it can be observed from Figure 7 that the IREP-2 scheme achieved a PSNR gain of 1 dB over the IREP-1 scheme and $E_{b}/N_{0}$ a gain of 0.6 dB as compared to the REP scheme at the PSNR degradation point of 1 dB. Figure 8 presents the subjective analysis results for the employed REP, IREP-1, and IREP-2 schemes for the transmission of the conceded “AKIYO” video sequence, from left-to-right, respectively for REP, IREP-1, and IREP-2 schemes. As observed from Figure 7, the PSNR performance of the IREP-2 scheme is better when compared to IREP-1 and REP; its effectiveness is visualized in the perceptual quality of the “AKIYO” video sequence, as shown in Figure 8.

## 7. Conclusions

This article presents a novel Irregular Error Protection (IREP) approach using Multi-code IRCC. This research aims to observe the convergence behavior of the iterative IRCC and the URC coding paradigm and exploit the innate irregular error protection capability of the Multi-code IRCC channel codes in combination with Unity Rate Coder (URC) while considering SP modulation aided DSTS transmitter diversity gain MIMO technique. The results were obtained while transmitting the H.264/AVC coded “KIYO” video phone sequence using REP, IREP-1, and IREP-2 error protected schemes. From the EXIT chart analysis of the REP, IREP-1, and IREP-2 error-protected schemes, it was observed that the iterative Multi-code IRCC coding paradigm in combination with URC results in better convergence behavior when compared to the using Uni-code IRCC employed in the REP scheme. Furthermore, improvement is achieved in the proposed system model by utilizing multidimensional SP modulation aided DSTS MIMO scheme. Additionally, a performance improvement is gained by exploiting the innate irregular error protection capability of the employed Multi-code IRCC, while keeping the relative importance of the H.264/AVC coded data partitions in view. Owing to the better convergence behavior of the employed irregular error protection scheme using Multi-code IRCC, it results in better BER performance in comparison with the regular error protection scheme using Uni-code IRCC. More specifically, it is observed that the employed IREP-2 scheme achieved an $E_{b}/N_{0}$, a gain of 1 dB with reference to the IREP-1 scheme, and an $E_{b}/N_{0}$, a gain of 0.6 dB as compared to the REP scheme, over the PSNR degradation of 1 dB. 

## Figures and Tables

**Table 1:** Qualitative analysis of the previous work and their contributions.

Year	Author	Contributions
1948	Shannon [12]	Established a relationship between block size, transmission rate, channel capacity, and error probability.
1950	Haming, R.W. [13]	Utilized first error detecting and single error correcting codes through redundancy
1952	Gilbert, E.N. [40]	Compared different signaling alphabets (Performance of signal alphabets is computed and it was concluded that complex alphabets are required in order to transmit a signal close to channel capacity.)
1955	Elias [14]	Presented Convolutional codes
1993	Barros et al. [15]	Presented turbo codes
1999	Ten Brink [21]	Proposed EXIT charts for analyzing the convergence behavior of iterative decoding and demapping
2002	Tucher and Hagenauer [24]	Proposed irregular convolutional codes as well as simplified method for computing mutual information in EXIT Chart analysis
2003	Weifeng Su et al. [8]	Proposed Sphere Packing (SP) modulation
2008	El-Hajjar et al. [10]	Proposed Differential Space Time Spreading (DSTS) codes
2016	Brejza et al. [32]	Implemented Fully Parallel Turbo Decoder (FPTD) on hardware design
2020	Minallah et al. [30]	Analyzed the convergence behavior of Self-Concatenated Convolutional Codes (SECCCs)
Proposed work	Minallah et al.	Presents the iterative convergence performance of IrRegular Convolutional Codes (IRCCs) with the aid of the multidimensional Sphere Packing (SP) modulation assisted Differential Space Time Spreading Codes (IRCC-SP-DSTS) Scheme.

**Table 2:** System parameters and their values.

Parametric Findings	Values
Source Compression Standard	H.264/AVC
Bit Rate	64 kbps
Per frame Slices	9
MBs Per Slice	11
MB Update Intra-Frame	3/frame
Channel Code	IRCC
Overall bit rate budget	0.7
Modulation Scheme	Sphere Packing
MIMO Scheme	DSTS
Transmitter Antennas	2
Receiver Antennas	1
Channel	Rayleigh fading (fd = 0.01)
Spreading Code	Walsh Code
Spreading Factor	4

**Table 3:** Error Protection Schemes and their Constituent Codes.

Error Protection Scheme	Order of Concatenation	Code Rate		
		Inner Code	Outer Code	Overall
Regular Error Protection (REP)	[A-B-C]	URC rate-1	IRCC Uni-code rate-3/4	3/4
Irregular Error Protection Scheme-1 (IREP-1)	[A-B-C]	URC rate-1	IRCC Multi-code rate-3/4	3/4
Irregular Error Protection Scheme-2 (IREP-2)	[B-A-C]	URC rate-1	IRCC Multi-code rate-3/4	3/4
